# Itch in Adult Population with Type 2 Diabetes Mellitus: Clinical Profile, Pathogenesis and Disease-Related Burden in a Cross-Sectional Study

**DOI:** 10.3390/biology10121332

**Published:** 2021-12-15

**Authors:** Aleksandra A. Stefaniak, Piotr K. Krajewski, Dorota Bednarska-Chabowska, Marek Bolanowski, Grzegorz Mazur, Jacek C. Szepietowski

**Affiliations:** 1 Department of Dermatology, Venereology and Allergology, Wroclaw Medical University, 50-368 Wroclaw, Poland; aleksandra.stefaniak@student.umed.wroc.pl (A.A.S.); piotr.krajewski@student.umed.wroc.pl (P.K.K.); 2 Department of Angiology, Hypertension and Diabetology, Wroclaw Medical University, 50-556 Wroclaw, Poland; dorota.bednarska-chabowska@umed.wroc.pl; 3 Department and Clinic of Endocrinology, Diabetes and Isotope Therapy, Wroclaw Medical University, 50-367 Wroclaw, Poland; marek.bolanowski@umw.edu.pl; 4 Department and Clinic of Internal Medicine, Occupational Diseases, Hypertension and Clinical Oncology, Wroclaw Medical University, 50-556 Wroclaw, Poland; grzegorz.mazur@umw.edu.pl

**Keywords:** diabetic itch, type 2 diabetes mellitus, pruritus

## Abstract

**Simple Summary:**

Although it is well accepted that itch might be one of the symptoms related to diabetes mellitus, very little is known about this topic from previous studies. In the current study, we aimed to investigate itch sensation in type 2 diabetes mellitus with special emphasis on the potential underlying causes. According to our study, itch was a relatively frequent symptom, present in about 36% of subjects, causing significant impairment to quality of life. Based on our findings and previous literature data, it seems that the primary cause of itch in this group of subjects is poor diabetes control with subsequent skin dryness and diabetic polyneuropathy.

**Abstract:**

Background: Despite growing interest in itch, data regarding itch in type 2 diabetes mellitus (DM2) are still limited, and mostly based on outdated studies. This study aimed to evaluate the clinical characteristics of itch in the adult population with DM2 and explore potential underlying causes. Methods: The study group consisted of 109 adult patients with DM2. Standardized questionnaires were completed in order to assess the itch intensity [Numerical Rating Scale (three days, 24hours) (NRS)] and the Four-item Itch Questionnaire (4IIQ) and to assess the psychological impact of itch [ItchyQoL, Six-Item Stigmatization Scale (6-ISS), Hospital Anxiety and Depression Scale (HADS)]. Skin dryness was evaluated clinically and by non-invasive assessment of epidermis moisturizing. Neuropathy was assessed using the clinical Katzenwadel neuropathy scale. Results: Itch occurred in 35.8% of adult patients with DM2, with NRS_max_ three days 6.31 ± 2.16 and 8.1 ± 3.5 points in 4IIQ. Itchy patients have had significantly higher FPG levels compared with the non-itchy population (*p* = 0.01). Patients with itch had a significantly higher possibility of neuropathy compared with non-itchy subjects (*p* < 0.01). Skin xerosis was significantly more advanced in patients with itch compared to those without (*p* < 0.01). The mean ItchyQol score was assessed as 41.2 ± 13.4 points, indicating mild life quality impairment and correlated positively with itch intensity. Itchy subjects had significantly higher scores in both anxiety and depression dimensions of HADS (in each *p* < 0.01). Conclusions: We suggest that the primary cause of itch is prolonged poor diabetes control with altered glucose and insulin levels, subsequently causing skin dryness and neuropathy in long-lasting DM2.

## 1. Introduction

Despite the growing interest in itch, data on itch in diabetes mellitus (DM) is rather limited and mostly based on outdated studies. Due to these reasons, physicians treating patients with DM may underestimate its frequency and clinical meaningfulness. DM constitutes a worldwide public health problem affecting 382 million people (8.3% of the world’s population) in 2013 with a projected increase to 592 million people (10.1%) in 2035 [1]. Pathogenesis of itch in DM is not fully understood, although in DM, the underlying pathophysiology, course of disease, comorbidities, complications and treatment used all tend to predispose patients to itch development. Type 2 diabetes mellitus (DM2) comprises most cases of all DM and is largely the result of excess body weight and physical inactivity [2].

Chronic Itch (CI) is defined as an unpleasant sensation that leads to intensive scratching lasting 6 weeks or longer [3]. According to the International Forum for the Study of Itch (IFSI), we may divide the etiologic classification of chronic itch to 6 categories: (I) dermatologic, (II) systemic, (III) neurologic, (IV) psychogenic/psychosomatic, (V) mixed, and (VI) others [4]. IFSI classifies itch in diabetes as systemic itch arising on non-inflamed skin or normal-appearing skin [5]. However, researchers have recently suggested that the etiology of itch in DM is additionally involved with both dermatologic and neurologic components [6,7,8]. The occurrence of itch in DM ranges from 18.4 to 27.5% [9]; however, the exact prevalence is not known, as most studies used inconsistent definitions and various tools for itch evaluation and included heterogeneous diabetic populations.

This study aimed to evaluate the prevalence of itch in the adult population of patients with DM2, using standardized methods for itch assessment, as well as to provide itch characteristics and to explore potential underlying causes.

## 2. Materials and Methods

### 2.1. Patients

This prospective cross-sectional study was performed between April 2019 and March 2021 with a break due to the COVID-19 pandemic situation (April 2020–September 2020). The project was approved by the local Ethical Committee (ST.C260.18.019). We approached 250 consecutive adult patients who were either treated due to diabetes in the University Hospital in Wroclaw or were patients treated in the outpatient unit, among whom 120 agreed to participate in the study (response rate: 48%). The inclusion criteria were diagnosis of DM2 according to internationally accepted criteria [10] and an informed written participation agreement. Exclusion criteria included: mental status changes deeming the patient unable to make a detailed assessment of itch, a history of chronic itch of another origin and treatment with antipruritic drugs (including drugs for peripheral neuropathy such as gabapentin and pregabalin). Among 120 patients, 10 subjects suffered from chronic itchy dermatological disorders (atopic dermatitis and psoriasis) and one patient was newly diagnosed with notalgia paresthetica. Therefore, the final study group consisted of 109 adult patients with DM2 (Table 1).

### 2.2. Study Design and Applied Questionnaires

After inclusion, detailed information on demographics, comorbidities, used treatment, and physical findings was obtained. The results of routine laboratory blood tests were also noted with particular emphasis on glycated haemoglobin (HbA1c) and fasting plasma glucose (FPG). FPG shows the glycemic control state at the time of the measurements, while the HbA1c is linked to average blood glucose levels in the past 7–8 weeks preceding the measurements. If multiple laboratory tests were available, the results closest to the day of dermatological examination have been chosen.

Standardized questionnaires and scales were completed by patients in order to assess the itch intensity [Numerical Rating Scale (three days, 24hours) (NRS; 0–10 points)] and the Four-item Itch Questionnaire [11,12,13] (4IIQ; 3–19 points). NRS cut-off points are as follows: 1– < 3 points represent a mild itch, 3–7 points a moderate itch, ≥7–9 points a severe itch, and ≥9 points very severe itch [14]. Furthermore, the description of cutaneous sensations associated with itch, factors alleviating, and aggregating itch, itch-related burden and sleep impairment were recorded.

Skin dryness was assessed clinically and with non-invasive corneometric assessment of epidermal hydration, using a Corneometer CM825, and neurological examination of the patient was done according to the guidelines of the American Diabetes Association [15], as previously described [12]. Based on neurological findings, all patients were assessed on the Katzenwadel neuropathy scale [16,17], where five aspects of polyneuropathy were assessed: movement functions, pain, sensorial function, motor coordination and tendon reflexes, which were assessed on a 3-point scale (range 0–2 points). The overall Katzenwadel neuropathy scale points correspond to the probability of clinically significant polyneuropathy as follows: with 0–2 points—low risk of neuropathy; 3–4 points—possible neuropathy; and 5–10 points—clinically visible neuropathy [16,17,18].

To assess the wide spectrum of psychological impact and itch-related burden, all itch patients were asked to complete the validated Polish language versions of an itch-specific quality of life questionnaire (ItchyQoL) and a Six-Item Stigmatization Scale (6-ISS) [17]. Regardless of experienced itching sensations, all patients were asked to fulfil the Hospital Anxiety and Depression Scale (HADS). ItchyQoL is the first questionnaire primary dedicated to itchy conditions and focuses not only on the impact of itch on daily activities but also on the characteristics of the symptoms and the experienced psychological burden level of psychological strain. It evaluates 3 dimensions of itch: symptoms, functioning and emotions [19]. ItchyQoL is also suitable for use in patients with itch without primary skin lesions. The ItchyQoL assessment consists of 22 questions, scored on a 5-point scale ranging from 1 (never) to 5 (all the time), with the sum forming the overall ItchyQol score (22–110 points). The overall ItchyQoL score corresponds to the level of itch-specific health-related quality of life (QoL) impairment as follows: 0–30 points (very mild); 31–50 points (mild); 51–80 points (moderate); and 81–110 points (severe impairment) [20]. The subscales (symptoms, functioning and emotions) are calculated as the mean scores pertaining to that particular category (range 1–5 points) [20]. For the assessment of stigmatization level (score range 0–18), 6-ISS was developed. The higher the scores, the greater the stigmatization level was observed [21]. HADS [22] is used as a self-evaluation rating scale of 14 items designed to measure anxiety and depression. It includes seven items assessing anxiety and seven for depression, each with a score of 0–3. Scores ranging from 0 to 7 are considered a normal case, from 8 to 10 a borderline case, and from 11 to 21 abnormal, and in need of further examination or requiring treatment.

### 2.3. Statistical Analysis

Statistical analysis of the obtained results was performed with the use of Statistica v. 12 (StatSoft, Kraków, Poland). All data was assessed for parametric or nonparametric distribution. The minimum, maximum, mean and standard deviation calculated. Quantitative variables were evaluated using the Mann–Whitney U test and Spearman’s and Pearson’s correlations. For qualitative data, the chi-squared test was used. Differences between more than two groups were assessed with the use of Kruskal–Wallis one-way analysis of variance on ranks. A 2-sided *p* value ≤ 0.05 was considered to be statistically significant

## 3. Results

### 3.1. General Demographics and Systemic Comorbidities

A total of 109 (56 females (51.4%); age range: 21–89 years; median age: 65 years) patients with DM2 were enrolled. The range of duration of diabetes was 0–44 years (mean duration: 12.8 ± 9.4; median: 12 years) (detailed characteristics of the study population is given in Table 1). Thirty-nine subjects reported itch during the course of DM2 (35.8%) while, at the time of examination (point prevalence), it was reported by 32.1% (*n* = 35) (Table 1). There was no significant difference between the analysed groups (itchy and non-itchy patients) concerning sex, age, body mass index (BMI), duration of the disease, level of education, smoking, drugs or alcohol intake behaviour. The most common systemic comorbidities encompassed cardiovascular disorders (63.3%), thyroid and parathyroid gland disorders (26.6%), and chronic kidney disease (11.9%), without significant differences between itch and non-itch patients. Regarding basic lab examinations, other than connected to glycemic control, there were no significant differences in glomerular filtration rate (eGFR) or serum creatinine and urea levels in patients with itch and without itch (detailed data not shown). All patients with known thyroid disease were euthyroid.

### 3.2. Clinical Characteristics of Itch

At the moment of examination, itch was present in 35 subjects out of 39 who experienced CI during the course of DM (89.7%). In this group of subjects, the maximal NRS score for the itch intensity during preceding three days was 6.3 ± 2.2 points (median: 6, range 3–10 points), while during the preceding 24 h was assessed as 4.9 ± 2.5 points (median: 5, range 1–10 points), indicating a moderate itch intensity. According to NRS, not even a single patient reported a mild itch. The moderate itch was observed in 54.29% of patients, while severe itch in 28.6% of patients and very severe itch was observed in 17.14%. The mean 4 IIQ score was 8.1 ± 2.5 points (median: 8, range: 3–16).

**Figure 1:**
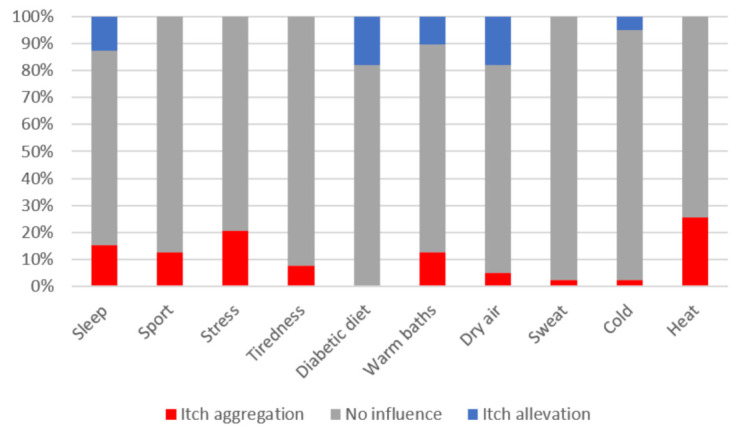
Factors exacerbating and relieving itch in patients with type 2 diabetes mellitus.

CI was usually localized, however, mostly in more than one area (51.3%). It mainly affected lower and upper limbs (38.5% and 23.1%), the trunk (30.8%) and the scalp (23.1%). Generalized itch bothered nine patients (23.1%), while anogenital itch affected only one participant (2.6%). Out of 24 patients with itch in limbs area, eight (40%) experienced itch in the area of hands and feet. Approximately 70% of patients (69.3%) suffered from itch on a daily basis, at least a few times a week. Most frequently, subjects described itch-related sensations as burning (38.5%, *n* = 15) and pinching (30.8%, *n* = 12), followed by tingling (28.2%, *n* = 11), stinging and tickling (in each sensation 12.8%, *n* = 5). Moreover, patients who suffered from itch reported this symptom as being predominantly burdensome (38.5%, *n* = 15), annoying (35.9%, *n* = 14), or unbearable or worrisome (in each 12.8%, *n* = 5). Although the itch sensation appeared most frequently in the afternoon, almost all of the patients reported itch also in other times of the day or at night (Table 2). About half of itchy participants had trouble falling asleep (almost always: 20.5%, occasionally: 30.8%) and 30.8% reported awakenings because of this symptom (almost always: 18%, occasionally: 12.8%). In addition, five (12.8%) subjects used medication for insomnia due to itch. Stress and heat were the most important factors exacerbating itch while sticking to diabetic diet was described as the factor relieving itch (Figure 1).

### 3.3. Factors Contributing to Itch in DM: Glycaemic Control, Polyneuropathy, Skin Xerosis, Treatment Used

**Figure 2:**
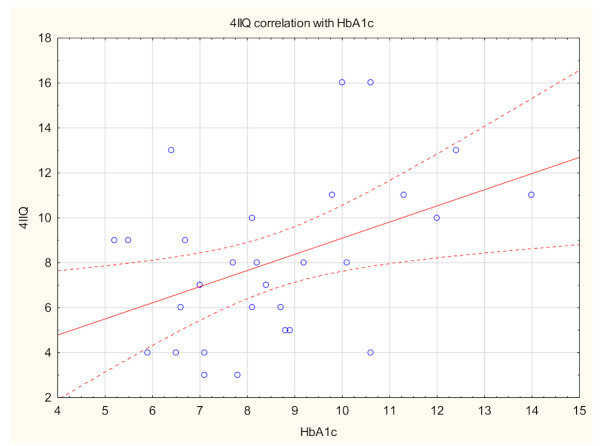
Spearman’s correlation analysis revealed a significant positive link between itch intensity measured with a Four-Item Itch Questionnaire (4IIQ) and glycated haemoglobin (HbA1c) values (R = 0.4, *p* = 0.05).

Itchy patients had significantly higher FPG levels compared with the non-itchy population (*p* = 0.01) (Table 3). Although there was no significant difference regarding HBA1C in Mann–Whitney U Test in disease populations, Spearman’s correlation analysis revealed a significant positive link between itch intensity measured with 4IIQ and HbA1c values (R = 0.4, *p* = 0.05) (Figure 2). Moreover, both HbA1c and FPG correlated positively with skin xerosis examined clinically (R = 0.4, *p* < 0.001, R = 0.2, *p* = 0.03, respectively), and polyneuropathy assessed in the Katzenwadel scale (R = 0.3, *p* = 0.01, R = 0.4, *p* < 0.01, respectively).

There were significant differences regarding the treatment of DM in itchy and non-itchy subjects. All patients, who received insulin treatment, received it subcutaneously. Patients with itch were prescribed insulin treatment significantly more often, compared to non-itchy group (*p* = 0.03) and patients on oral treatment usually did not experience itch (*p* < 0.01) (Table 3). Moreover, metformin treatment was significantly more common in non-itchy subjects (*p* = 0.01). There were no significant differences regarding other groups of oral medications used (sulfonylurea derivatives [gliclazide, glimepiride], acarbose, dipeptidyl peptidase 4 inhibitors, sodium-glucose transport protein 2 inhibitors, glucagon-like peptide-1 receptor agonists) by our patients and itch (detailed data not shown).

Patients with itch usually also reported other sensations, such as tingling, numbness, stinging or burning, clinically connected to polyneuropathy (*p* < 0.01) (details shown in Table 3). The Mann–Whitney U Test revealed that itchy subjects had a significantly higher probability of neuropathy (in Katzenwadel’s neuropathy scale). The possibility of clinically relevant neuropathy was diagnosed in about 50% of itchy patients (possible neuropathy in 35.6%, *n* = 14, clinically visible neuropathy in 15.4%, *n* = 6), while in the non-itchy group it was diagnosed in less than quarter (possible neuropathy in 22.9%, *n* = 16, clinically visible neuropathy in 2.9%, *n* = 2). Patients without itch had a significantly lower possibility of neuropathy compared with itchy subjects (*p* < 0.01), while clinically visible neuropathy was more frequent in the itchy group (*p* = 0.01). Patients with longer duration of the disease had higher risk of neuropathy (R = 0.4, *p* < 0.01). Patients with possible neuropathy according to Katzenwadel’s neuropathy scale have felt also significantly more frequently sensations such as tingling (*p* < 0.01), numbness (*p* < 0.01) or burning (*p* < 0.01). Sensation of any of these feelings (tingling, pain, burning, numbness, stinging, hyperesthesia or hypoesthesia) was connected to higher probability of polyneuropathy (*p* < 0.01). Polyneuropathy was, however, not connected to generalized nor localized itch. The area affected with itch was not connected to the neuropathy either (detailed data not shown). Other factors (age, smoking packet year, BMI) were not linked to the possibility of polyneuropathy in our group of patients.

**Figure 3:**
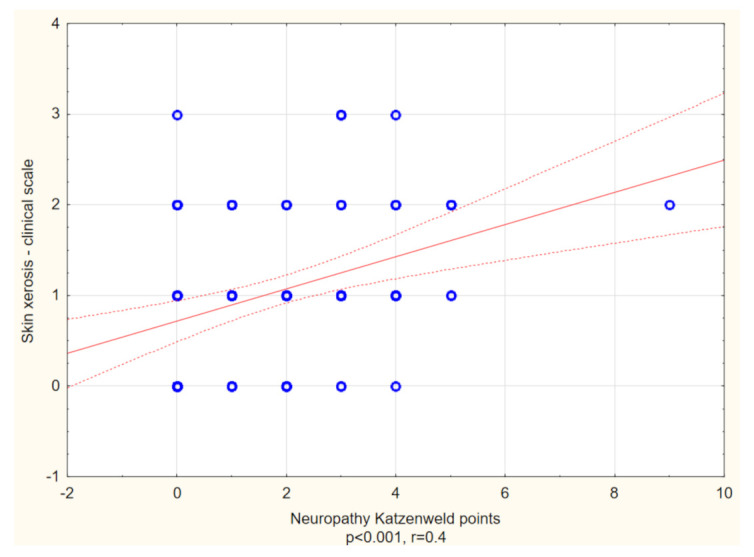
Spearman’s correlation analysis revealed a significant positive link between skin xerosis assessed in clinical scale and neuropathy assessed with Katzenwadel neuropathy scale (R = 0.4, *p* < 0.001).

Skin xerosis, examined clinically, was significantly more advanced in patients with itch compared with those without (*p* < 0.01), although using corneometry, there was no difference between these groups with regard to epidermal hydration values (Table 3). However, Spearman’s correlation analysis revealed a significant positive link between skin xerosis examined clinically and neuropathy assessed in Katzenwadel ‘s neuropathy scale (R = 0.4, *p* < 0.01) (Figure 3). What is interesting, in the corneometric assessment in the area of the thorax, is that skin hydration was lower in patients with longer duration of DM2 (R = −0.2, *p* = 0.02) and with higher probability of neuropathy (R = −0.2, *p* = 0.04). Other factors positively associated with skin dryness assessed clinically were age (R = 0.2, *p* = 0.01) and smoking packet years (R = 0.4, *p* < 0.01). Additionally, in patients with higher BMI the skin was dryer in the abdominal area in corneometrical assessment (R = −0.3, *p* < 0.01).

### 3.4. Itch and Patients Well-Being

**Figure 4:**
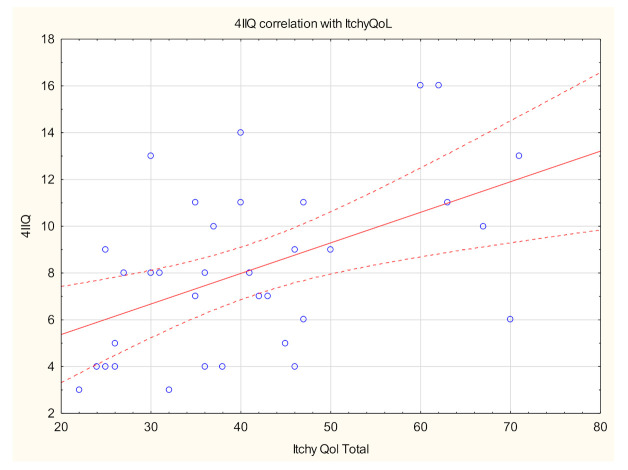
Spearman’s correlation analysis revealed a significant positive link between itch intensity measured with Four-Item Itch Questionnaire (4IIQ) and life impairment due to itch measured with ItchyQoL (itch-specific quality of life questionnaire) (R = 0.5, *p* < 0.01).

The mean ItchyQol score was assessed as 41.2 ± 13.4 points, indicating mild itch-related QoL impairment (Table 4). Itch-related impairment of QoL ranged from very mild to moderate, with nearly a quarter (23.7%) indicating very mild, more than half (55.3%) mild and 18.4% moderate impact. One patient refused to fill out this questionnaire. A positive correlation between the intensity of itch, estimated with 4IIQ, and ItchyQol was found (R = 0.5; *p* < 0.01) (Figure 4). With reference to the results for the subscales, itch in DM2 had the highest impact on symptoms dimension, while emotion and functioning were quite equally scored (Table 5). Itch intensity measured with 4IIQ was also linked with symptoms and functioning dimensions (R = 0.5, *p* < 0.01, R = 0.5, *p* = 0.01, respectively).

All thirty-five patients with itch during the preceding three days fulfilled the 6-ISS questionnaire. The mean score was 1.5 ± 1.8 points in this cohort of our patients (Table 4). There was also no significant relationship between itch intensity and 6-ISS scores.

**Figure 5:**
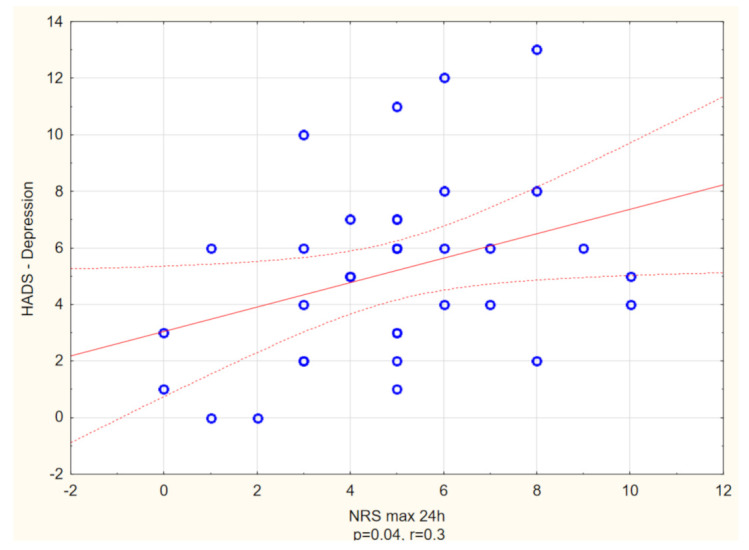
Spearman’s correlation analysis revealed a significant positive link between maximal itch intensity measured with Numerical Rating Scale (NRS) and possibility of clinically relevant depression measured with Hospital Anxiety and Depression Scale (HADS) (R = 0.3, *p* = 0.04).

**Figure 6:**
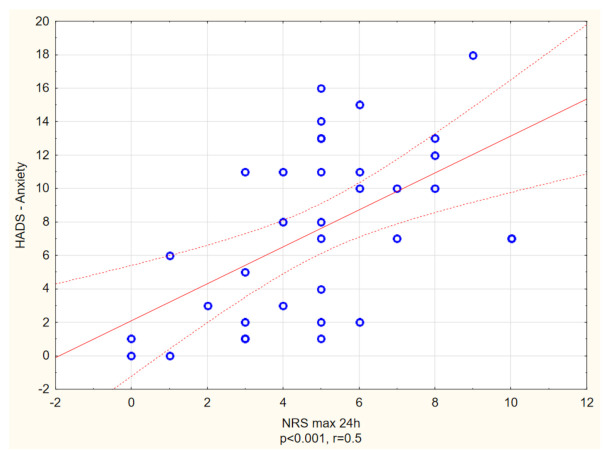
Spearman’s correlation analysis revealed a significant positive link between maximal itch intensity measured with Numerical Rating Scale (NRS) and level of anxiety measured with Hospital Anxiety and Depression Scale (HADS) (R = 0.5, *p* < 0.001).

With reference to HADS, the prevalence of anxiety and depression among itchy patients with DM2 was estimated as 33.3% and 7.6%, respectively, while in the non-itchy group it was 5.7% (anxiety) and 1.4% (depression). Itchy subjects had significantly higher scores in both anxiety and depression dimensions of HADS (in each *p* < 0.01) (Table 4). Moreover, the itch intensity measured in NRS (24h) correlated positively with both HADS depression, as well as HADS anxiety scores (R = 0.3, *p* = 0.04, R = 0.5, *p* < 0.01, respectively) (Figure 5 and Figure 6).

Sex, age, BMI, duration of the disease did not correlate with any of the dependencies in the utilized questionnaires.

## 4. Discussion

Although the first known interest in itch in DM was in the late 1920s [23], until now, no other study or review, to our knowledge, has comprehensively investigated and characterized itch in the adult population with DM2. We found high prevalence of itch in this group of patients (35.8%), with a higher rate than reported in the majority of available studies involving patients suffering from DM (18.4–27.5%) [8,24,25]. Itch in DM2 may be both localized, affecting mainly lower extremities (38.5%), trunk (30.8%) and scalp (23.1%), as well as generalized (23.1%). According to literature, generalized itch was noted in 20–27.5% of the patients [25,26], which is in concordance with our study. Valdes-Rodriguez et al. [27] found localized itch in the scalp area in about 40% of elderly patients with DM2. Higher estimated prevalence reported in the study by Valdes-Rodriguez et al. might be connected to the fact that patients were not screened for other possible causes of itch. Moreover, anogenital itch, commonly known in the case of women as pruritus vulvae, was also a subject of studies during the years, with estimated prevalence in DM as 19–27.2% [28,29]. In our study only one subject reported anogenital pruritus (2.6%). Pruritus vulvae might be a result of vaginal infection, which is more common in poorly controlled DM. In our study we excluded patients with ongoing infections, as infections might become independent itch revelators [30]. Moreover, in our study only patients with itch lasting longer than six weeks were qualified, and that is probably why, in our patients, this prevalence was lower. Comparing to our previous study [12], which described itch in the pediatric population with type I diabetes mellitus (DM1), the prevalence of itch in the adult population was higher (22% in DM1 vs. 35.8% in DM2). The distribution of itch also differed, just as in pediatric patient itch, where it was usually localized in upper and lower extremities (68.2% and 50%, respectively). None of the affected subjects with DM1 reported generalized itch. Itch intensity in our study is in concordance with that previously reported in the pediatric population with DM1 (Table 6). In both studies, a diabetic diet was reported by patients as an itch-alleviating factor.

Pathogenesis of itch in DM is not fully understood and various factors are described as contributing to the development of this symptom. Currently, the researchers believe that there are two main factors associated with the itch in diabetes—skin xerosis and diabetic polyneuropathy, indicating a dermatological or neurological origin of itch. The role of glycemic control is still being discussed. Based on our study it seems that all of these factors contribute to itch pathogenesis in the adult population with DM2. On the cellular level, insulin is an essential growth factor for cultured keratinocytes and it influences keratinocyte proliferation, migration and differentiation [31]. Additionally, according to recent studies, increased oxidative stress and nerve inflammation may also play a role in diabetic polyneuropathy [32]. One of the theories suggests that atypical changes in insulin levels in the circulating blood might be the cause of the disrupted keratinocytes. Therefore, abnormal proliferation of keratinocytes in the epidermis changes the functions of stratum corneum in patients with diabetes. Moreover, it is well known that DM induces an increase in advanced glycosylation products in the collagen of the dermis, that may be connected to skin ageing [33]. All of this may be the cause of the reduced hydration of the stratum corneum [34,35] and, subsequently, cause itch. In the current study we have shown that both HbA1c and FPG correlated positively with skin xerosis examined clinically. In our study on the pediatric population with DM1 [12], where mean duration of DM was less than six years, skin xerosis was the most important factor influencing itch presence and intensity. In the current study, patients with itch had significantly dryer skin, compared with non-itchy individuals. Newer theories suggest that itch in DM may be associated with diabetic polyneuropathy. Neuropathy or damage of the nerve fibres is a debilitating, yet surprisingly common and complex, condition. By far, DM is the most common cause of neuropathy in developed countries [36,37] with the estimated prevalence of 30%, and while up to 50% of patients will eventually develop some kind of neuropathy during the course of the disease [38]. DM may cause the damage to the peripheral nervous system in a variety of ways, but the most common presentation is a distal symmetric polyneuropathy (DSP) followed by small fiber predominant neuropathy (SFN), radiculoplexopathy and autonomic neuropathy [39]. SFN is considered an early manifestation of diabetic polyneuropathy and is known to progress to distal symmetric polyneuropathy [40]. Typical clinical manifestations of diabetic polyneuropathy (numbness, tingling, neuropathic pain, hyper and hypoesthesia and/or weakness, stocking-and-gloves distribution of symptoms, risk of ulcerations) are considered to be manifestations of DSP. SFN is a disorder of the small myelinated Aδ-fibers and unmyelinated C-fibers and it might affect small sensory fibers, autonomic fibers or both, resulting in sensory changes, autonomic dysfunction, or combined symptoms. SFN is more difficult to diagnose, as patients rarely show decreased reflexes, weakness, and impaired vibration sensations—assets which are typically screened to asses diabetic polyneuropathy. In most patients, different kinds of neuropathies come together, and are mainly a result of the long-lasting hyperglycemia, with created changes in insulin signalling as a key. The theory of itch in DM is mostly focused on itch as a result of both DSP and SFN, and is as closely related to skin xerosis, as diabetic polyneuropathy is linked to the dysfunction of sweating, which is due to impairment of the sympathetic nervous system [41]. Pereira et al. [42], in their study concerning itch in SFN, showed that in about 18% of cases, neuropathy was caused by DM. In our study, we have shown that both HbA1c and FPG correlated positively with polyneuropathy assessed with the Katzenwadel scale. Furthermore, we have provided a clear link between skin xerosis examined clinically and polyneuropathy. What is interesting, in corneometric assessment in the area of the thorax, is that skin hydration was lower in patients with longer duration of DM2 and in subjects with higher probability of neuropathy. In our opinion, this observation may explain the pathogenesis of truncal pruritus of unknown origin (TPUO) in diabetic patients. Yamaoka et al. [8], in 2010, first documented a clear link between TPUO and DM2. As a result of a multicentre study on 391 diabetic outpatients, Yamaoka et al. [8] proved that prevalence of TPUO in diabetic subjects was significantly higher than that in age-matched nondiabetic subjects (11.3 vs. 2.9%, *p* = 0.0001). In our study, besides itching, patients usually also reported other sensations, such as tingling, numbness, stinging or burning, clinically connected to SFN and neuropathic itch [42,43]. Patients without itch had significantly lower possibility of neuropathy compared with itchy subjects, while clinically visible neuropathy was more frequent in the itchy group. Therefore, we would like to suggest itch as one of the sensations connected to diabetic polyneuropathy.

**Figure 7:**
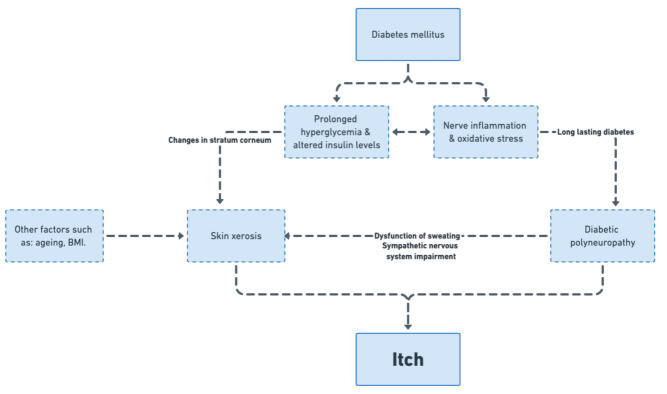
Proposed pathogenetic model of itch in diabetes.

Regarding glycemic control, in our cohort, itchy patients had significantly higher FPG levels compared with the non-itchy population. Additionally, we have provided significant positive association between itch intensity measured with 4IIQ and HbA1c values. There is a paucity of literature regarding itch and parameters of glycemic control. Ko et al. [25] showed that patients with a higher postprandial glucose level had a higher probability of generalized itch. Hillson et al. [44] documented the correlation between the FPG and generalized itch in newly diagnosed and untreated patients with DM2. In patients with short-lasting DM1 [12] there was no association between itch and glycemic control, however, nearly a quarter (22.7%) considered a diabetic diet as itch-alleviating, as in this currently described study on adult population with DM2. Several antidiabetic medications (glimepiride, metformin, tolbutamide) were also associated with CI [30]. In our study, patients treated with oral medications showed lower probability of itch, compared to patients receiving insulin. Nota bene, no association was found between type of oral treatment and itch. We may only speculate but, in our opinion, this is linked to the control of the disease as well. Patients with need of insulin treatment usually cannot achieve proper disease control on oral treatment only. Based on these findings, we would like to suggest a model of itch in DM (Figure 7) with glycemic control as the main factor contributing to both skin xerosis and diabetic polyneuropathy. Based on literature and our findings, it is possible that in short-lasting DM the dermatological component plays a bigger role, while longer duration of the disease may contribute to neuropathic co-factors. Furthermore, more advanced age and comorbidities frequently associated with DM, such as metabolic syndrome and chronic kidney disease, may contribute to the dermatological component—skin dryness. Therefore, we would like to emphasize that, in our opinion, itch in diabetes should be classified as systemic itch, according to IFSI classification [4], with both dermatological and neurological components as co-factors.

In our cohort of patients, itch correlated positively with impairment of QoL as measured with ItchyQoL, both in total, as in the symptom and functioning subscores. This corresponds with other studies on both cutaneous and systemic itch and QoL assessment [45,46,47]. In general, it is known that CI is a highly debilitating symptom in the majority of itch patients. Moreover, itchy subjects had significantly higher scores in both anxiety and depression dimensions of HADS. As the HADS questionnaire is commonly used worldwide, it is quite easy to conduct an assessment comparing scores in various itchy disorders. For instance, the HADS results obtained in our study showed comparable deterioration to systemic itch in polycythaemia vera [48], as well as some other itchy skin disorders, including psoriasis [49], atopic dermatitis [50], urticaria [51,52], chronic prurigo [53] or acne [54], known to cause relevant disability.

This study has several limitations. Firstly, this was a single-centre study and the number of subjects with itch was relatively small, therefore, this study should be considered of an exploratory nature. In addition, the descriptive nature of the study must be considered. Secondly, insulin and sorbitol levels were not measured and there was no differentiation in the types of insulin used by the patient. Additionally, as patients with poor diabetes control and longer duration of the disease are known to have greater risk for complications, possible risk of multicollinearity have to be taken into consideration. Last, but by no means least, the period of the study was relatively long, with a break due to the COVID-19 pandemic. Despite our efforts, additional factors influencing both itch and patient well-being could affect the results.

## 5. Conclusions

In conclusion, itch in DM2 is a complex symptom, arising relatively frequently. Based on literature and our findings, we suggest that the primary cause of itch is prolonged poor diabetes control with altered glucose and insulin levels, subsequently causing skin dryness and small-fibre neuropathy in long-lasting DM2. Proper control of diabetes should be the main goal of treatment of itch in this group of patients, however the dermatological and neuropathic component should not be neglected. Moreover, we would like to suggest itch as one of the symptoms of diabetic polyneuropathy.

## Figures and Tables

**Table 1:** Characteristics of the studied population.

	Total	With Itch	Without Itch	*p*-Value *
Sex (*n*) Female Male	109 56 (51.4%) 53 (48.6%)	39 24 (61.5%) 15 (38.5%)	70 32 (45.7%) 38 (54.3%)	NS
Age (years), mean ± SD Range Median	62.4 ± 14.4 (21–89) 65	60.3 ± 13.8 (23–83) 65	63.5 ± 14.7 (21–89) 65	NS
BMI (kg/m^2^), mean ± SD Range Median	29.4 ± 5.7 (18.7–48.4) 28.2	29.7 ± 6.1 (18.7–48.4) 29.1	29.2 ± 5.4 (19.1–46.0) 28.1	NS
Duration of diabetes (years), mean ± SD Range Median	12.7 ± 9.5 (0–45) 12	14 ± 8.9 (0–32) 13	12 ± 12.7 (0–45) 10	NS
Level of education (*n*) Primary Secondary Higher education No data	18 (16.5%) 49 (45%) 24 (22%) 18 (16.5%)	7 (18%) 18 (46.1%) 9 (23.1%) 5 (12.8%)	11 (15.7%) 31 (44.3%) 15 (21.4%) 13 (18.6%)	NS NS NS
Stimulant use Smoking Alcohol Drugs	48 (44%) 30 (27.5%) 3 (2.8%)	20 (51.3%) 13 (33.3%) 2 (5.1%)	28 (40%) 17 (24.3%) 1 (1.4%)	NS NS NS
Packet years (years), mean ± SD Range Median	19.8 ± 19.4 (0–90) 15	18.1 ± 15.2 (0–50) 10	21 ± 21.9 (0–90) 12.5	NS
Comorbidities:				
Cardiovascular disorders	69 (35.8%)	22 (56.4%)	47 (67.1%)	NS
Thyroid and parathyroid gland disease	29 (26.6%)	12 (30.8%)	17 (58.6%)	NS
Chronic kidney disease	13 (11.9%)	6 (15.4%)	7 (10%)	NS
Malignancies in the past and neoplasms (incl. benign prostatic hyperplasia)	10 (9.2%)	5 (12.8%)	5 (7.1%)	NS
Joint diseases	5 (4.6%)	1 (2.6%)	4 (5.7%)	NS
Asthma and COPD	4 (3.7%)	1 (2.6%)	3 (4.3%)	NS
Neurological disorders	4 (3.7%)	1 (2.6%)	3 (4.3%)	NS
Infectious diseases in the past (incl. borreliosis, syphilis, meningitidis, tuberculosis)	4 (3.7%)	2 (5.1%)	2 (2.9%)	NS
Psychiatric disorders	4 (3.7%)	2 (5.1%)	2 (2.9%)	NS
Gastrointestinal disorders	3 (2.7%)	0 (0%)	3 (4.3%)	NS

SD—standard deviation, BMI—Body Mass Index, NS—not significant, COPD—chronic obstructive pulmonary disease, HADS—Hospital Anxiety and Depression Score, * Group with itch vs. group without itch.

**Table 2:** Occurrence of pruritus during different times of the day.

Time of the Day/Frequency	Not at All	Rarely	Often	All the Time
Morning	8 (20.5%)	24 (61.6%)	5 (12.8%)	2 (5.1%)
Afternoon	1 (2.6%)	22 (56.4%)	13 (33.3%)	3 (7.7%)
Evening	8 (20.5%)	20 (51.3%)	9 (23.1%)	2 (5.1%)
Night	9 (23.1%)	17 (43.6%)	12 (30.7%)	1 (2.6%)

**Table 3:** Studied etiopathogenetic factors contributing to itch in diabetes mellitus and dependencies on selected clinical data.

	With Itch (*n* = 39)	Without Itch (*n* = 70)	*p*-Value
Glycaemic control:			
HbA1C (%), mean ± SD Range Median	8.5 ± 2 (5.2–14) 8.2	8 ± 2.3 (5–15.1) 7.5	NS
FPG (mg/dl), mean ± SD Range Median	174.6 ± 62.3 (88–356) 164	148 ± 69.2 (62–510) 124	*p* = 0.01
Treatment of diabetes: (*n*)			
Insulin	17 (43.6%)	18 (25.7%)	*p* = 0.03
Oral treatment	20 (51.3%)	56 (80%)	*p* < 0.01
Diet only	3 (7.7%)	1 (1.4%)	NS
Skin xerosis:			
Skin xerosis examined clinically (points), mean ± SD Range Median	1.3 ± 0.8 (0–3) 1	0.9 ± 0.9 (0–3) 1	*p* < 0.01
Epidermal hydration (AU), mean ± SD (median) • Forearm • Lower leg • Abdomen • Chest	27.4 ± 10.6 (28.9) 32.1 ± 13.2 (33.3) 20.4 ± 9.4 (18.9) 39.4 ± 14.6 (38.2)	31.2 ± 12.2 (29.9) 32.8 ± 12.4 (31.6) 25.9 ± 16.2 (21.3) 37.1 ± 18.5 (39.3)	NS NS NS NS
Polyneuropathy:			
Other than itch sensations related to polyneuropathy (*n*): • Tingling • Numbness • Pain • Stinging • Burning • Hyperesthesia • Hypoesthesia	26 (66.7%) 22 (56.4%) 8 (20.5%) 7 (17.9%) 14 (35.9%) 7 (17.9%) 4 (10.3%)	20 (28.6%) 22 (31.4%) 8 (11.4%) 3 (4.3%) 7 (6.4%) 7 (6.4%) 2 (2.9%)	*p* < 0.01 *p* = 0.01 NS *p* = 0.02 *p* < 0.01 NS NS
Katzenwadel scale (points), mean ± SD Range Median	3.0 ± 1.8 (0–9) 3	1.3 ± 1.4 (0–5) 1	*p* < 0.01

HbA1C—glycated haemoglobin, FPG—fasting plasma glucose, SD—standard deviation.

**Table 4:** Questionnaires’ scores and dependencies on selected clinical data.

	With Itch (*n* = 39)	Without Itch (*n* = 70)	*p*-Value
HADS-Anxiety (points), mean ± SD Range Median	7.6 ± 4.9 (0–18) 7	3.9 ± 3.9 (0–21) 3	*p* < 0.01
HADS-Depression (points), mean ± SD Range Median	5.1 ± 3 (0–13) 5	3 ± 3.3 (0–21) 3	*p* < 0.01
6-ISS, mean ± SD Range Median	1.5 ± 1.8 (0–8) 1	-	NA
ItchyQoL (raw), mean ± SD Range Median	41.2 ± 13.4 (22–71) 39	-	NA

HADS—Hospital Anxiety and Depression Scale; 6-ISS—Six-Item Stigmatization Scale; ItchyQoL— itch-specific quality of life questionnaire; NA—not applicable.

**Table 5:** ItchyQoL subscale results among patients with itch in type 2 diabetes mellitus.

ItchyQol Subscale Mean Scores ± SD (Median)	
Symptoms	2.1 ± 0.8 (2.1)
Functioning	1.7 ± 0.6 (1.7)
Emotions	1.8 ± 0.8 (1.6)
Combined	1.9 ± 0.6 (1.8)

ItchyQoL—itch-specific quality of life questionnaire.

**Table 6:** Itch intensity in adult population with type 2 diabetes mellitus compared to pediatric population with type I diabetes mellitus [16].

	NRS_max_ Three Days Mean ± SD	NRS_max_ 24 h Mean ± SD	4IIQ Mean ± SD
Adult population with DM2 (current study)	6.31 ± 2.16	4.9 ± 2.5	8.1 ± 3.5 points
Pediatric population with DM1 (Stefaniak et al. 2020)	6.3 ± 3.0	5.0 ± 3.8	6.7 ± 3.5 points

NRS: Numerical Rating Scale, 4IIQ: Four-Item Itch Questionnaire, DM1: type I diabetes mellitus, DM2: type 2 diabetes mellitus.

## Data Availability

The datasets generated and analyzed in the current study are available from the corresponding author on reasonable request.
